# Supplementary material for: Artificially cloaked viral nanovaccine for cancer immunotherapy
Source: Nat Commun. 2019 Dec 17;10:5747. doi: 10.1038/s41467-019-13744-8 (PMC6917704; doi:10.1038/s41467-019-13744-8)
Supplement: Supplementary file 1 — Supplementary Information [file 41467_2019_13744_MOESM1_ESM.pdf]

# Supplementary Information

## Artificially cloaked viral nanovaccine for cancer immunoptherapy

Manlio Fusciello[1,†], Flavia Fontana[2,†], Siri Tähtinen[1], Cristian Capasso[1], Sara Feola[1], Beatriz Martins[1], Jacopo Chiaro[1], Karita Peltonen[1], Leena Ylösmäki,[1] Erkko Ylösmäki[1], Firas Hamdan[1], Otto K. Kari[1], Joseph Ndika[3], Harri Alenius[3,4], Artto Urtti [1,5], Jouni T. Hirvonen[2], Hélder A. Santos[2,6,*], Vincenzo Cerullo[1,6,7*]

[1] Drug Research Program, Division of Pharmaceutical Biosciences, Faculty of Pharmacy, University of Helsinki, FI-00140, Helsinki, Finland

[2] Drug Research Program, Division of Pharmaceutical Chemistry and Technology, Faculty of Pharmacy, University of Helsinki, FI-00140, Helsinki, Finland

[3] Systems Toxicology Group, Department of Bacteriology and Immunology, Medicum, University of Helsinki, FI-00140, Helsinki, Finland

[4] Institute of Environmental Medicine, Karolinska Institutet, SE-17177, Stockholm, Sweden

[5] School of Pharmacy, Faculty of Health Sciences, University of Eastern Finland, FI-70211, Kuopio, Finland

[6] Helsinki Institute of Life Science (HiLIFE), University of Helsinki, FI-00014, Helsinki, Finland.

[7] Department of Molecular Medicine and Medical Biotechnology and CEINGE, Federico II University of Naples, 80131, Naples, Italy

*Corresponding authors: helder.santos@helsinki.fi vincenzo.cerullo@helsinki.fi.

[†]These authors contributed equally to this work.

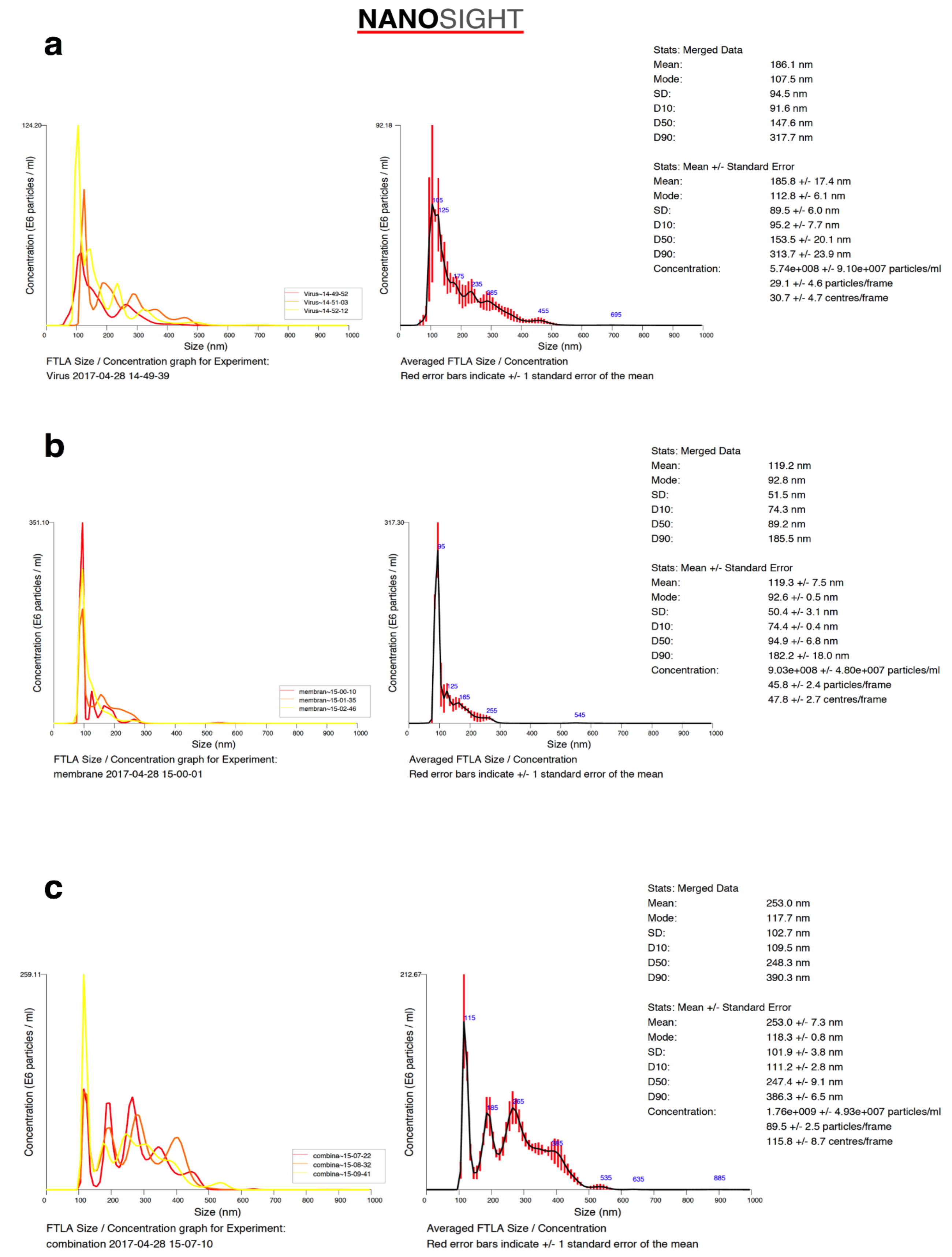


**Supplementary Figure 1. NTA analyses reports and characterization of the systems. (a)** Naked virus, **(b)** cancer membrane vesicles, and **(c)** ExtraCRAd.

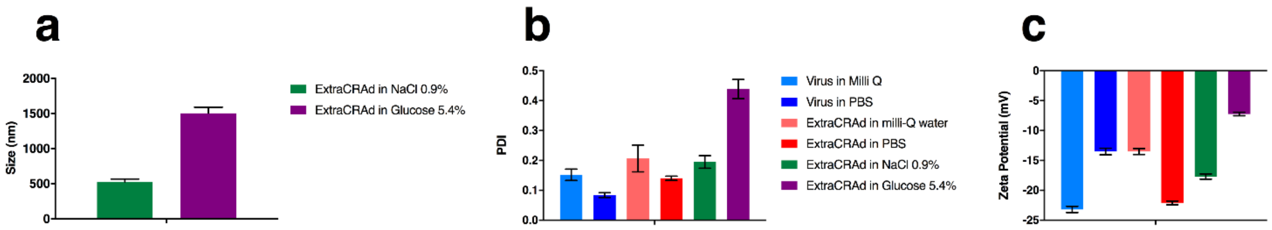


**Supplementary Figure 2. (a)** Size (nm) of ExtraCRAd extruded in 0.9% of saline solution or 5.4% of glucose solution. (**b)** Polydispersity index (PdI) of the virus before and after extrusion in the different buffers. **(c)** Surface charge of the virus before and after extrusion in different buffers. PBS, milli-Q water, 0.9% of NaCl saline solution and 5.4% of glucose isotonic solutions were evaluated as buffers in the formulation of our system. PBS solution showed the best results in terms of sample homogeneity and surface charge, thereby was selected for future applications. The results are presented as mean±s.d. (*n=3*).

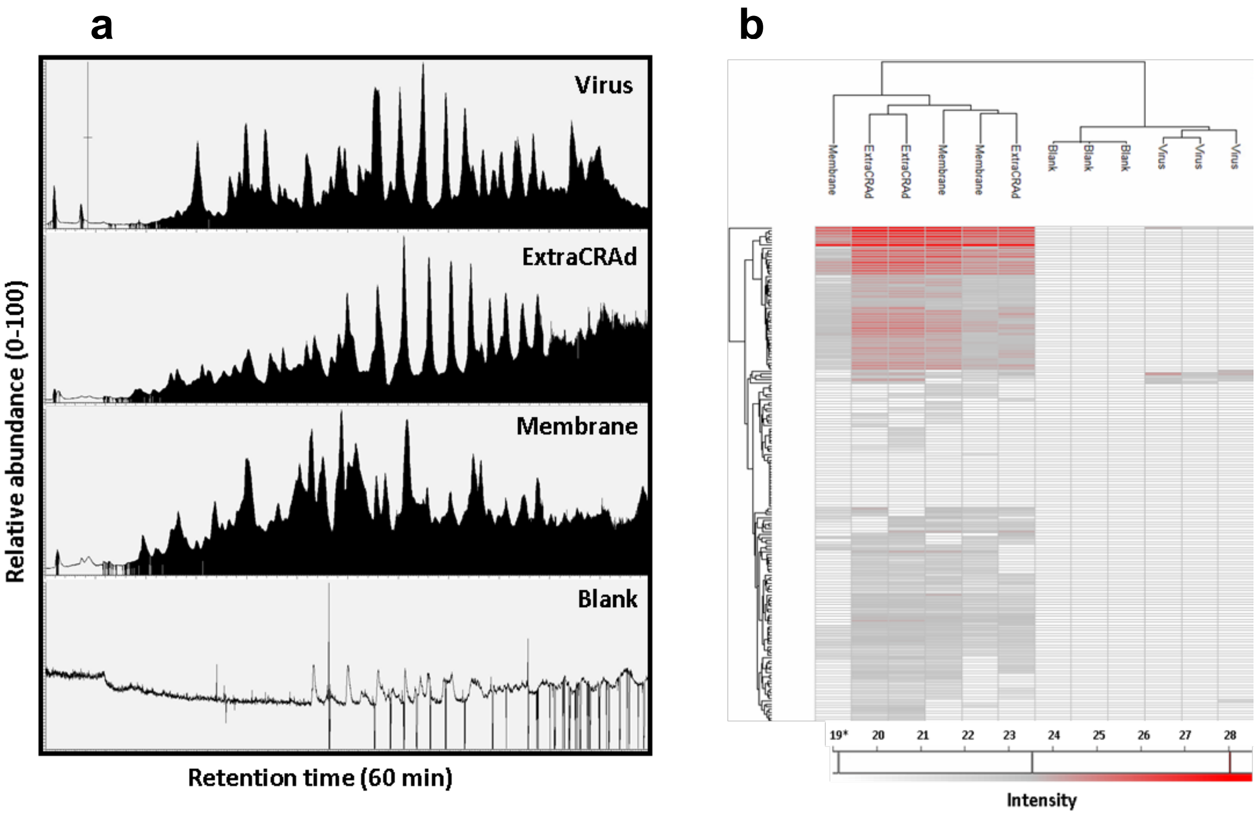


**Supplementary Figure 3. (a)** Representative chromatographic profiles highlighting the presence of tryptic peptides in virus, membrane, ExtraCRAd and no peptides in the Blank sample. **(b)** Hierarchical cluster of identified human proteins depicting little to no enrichment of human cell membrane proteins in virus and blank samples respectively, as opposed to the ExtraCRAd fraction.

7 Virus

| Index | Name | P-value | Adjusted p-value |
|---|---|---|---|
| 1 | Pathogenic Escherichia coli infection_Homo sapiens_hsa05130 | 6.834e-7 | 0.00001845 |
| 2 | Phagosome_Homo sapiens_hsa04145 | 0.00001532 | 0.0002068 |
| 3 | Gap junction_Homo sapiens_hsa04540 | 0.0003962 | 0.003566 |

142 ExtraCRAd

| Index | Name | P-value | Adjusted p-value |
|---|---|---|---|
| 1 | Carbon metabolism_Homo sapiens_hsa01200 | 9.495e-24 | 1.757e-21 |
| 2 | Glycolysis / Gluconeogenesis_Homo sapiens_hsa00010 | 7.872e-14 | 7.282e-12 |
| 3 | Biosynthesis of amino acids_Homo sapiens_hsa01230 | 2.755e-13 | 1.699e-11 |
| 4 | Metabolic pathways_Homo sapiens_hsa01100 | 1.003e-12 | 4.640e-11 |
| 5 | Ribosome_Homo sapiens_hsa03010 | 1.886e-12 | 6.979e-11 |
| 6 | Pyruvate metabolism_Homo sapiens_hsa00620 | 1.244e-11 | 3.837e-10 |
| 7 | Protein processing in endoplasmic reticulum_Homo sapiens_hsa04141 | 3.326e-11 | 8.790e-10 |
| 8 | Citrate cycle (TCA cycle)_Homo sapiens_hsa00020 | 4.001e-11 | 9.252e-10 |
| 9 | Antigen processing and presentation_Homo sapiens_hsa04612 | 5.812e-9 | 1.195e-7 |
| 10 | Pathogenic Escherichia coli infection_Homo sapiens_hsa05130 | 7.113e-9 | 1.316e-7 |

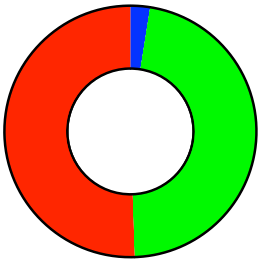

132 Membrane

| Index | Name | P-value | Adjusted p-value |
|---|---|---|---|
| 1 | Carbon metabolism_Homo sapiens_hsa01200 | 5.725e-23 | 1.025e-20 |
| 2 | Glycolysis / Gluconeogenesis_Homo sapiens_hsa00010 | 2.844e-14 | 2.546e-12 |
| 3 | Metabolic pathways_Homo sapiens_hsa01100 | 3.557e-13 | 2.122e-11 |
| 4 | Biosynthesis of amino acids_Homo sapiens_hsa01230 | 3.002e-12 | 1.343e-10 |
| 5 | Pyruvate metabolism_Homo sapiens_hsa00620 | 5.814e-12 | 2.082e-10 |
| 6 | Citrate cycle (TCA cycle)_Homo sapiens_hsa00020 | 2.033e-11 | 6.065e-10 |
| 7 | Protein processing in endoplasmic reticulum_Homo sapiens_hsa04141 | 1.518e-10 | 3.882e-9 |
| 8 | Ribosome_Homo sapiens_hsa03010 | 2.639e-9 | 5.904e-8 |
| 9 | Pathogenic Escherichia coli infection_Homo sapiens_hsa05130 | 9.358e-8 | 0.000001861 |
| 10 | Antigen processing and presentation_Homo sapiens_hsa04612 | 9.785e-7 | 0.00001751 |

**Supplementary Figure 4. KEGG Pathway analysis of enriched proteins from the specified fractions.** Identified proteins represent RNA binding ribosomal proteins (virus, ExtraCRAd and membrane), antigen processing/presentation, as well as cell metabolism (ExtraCRAd and membrane).

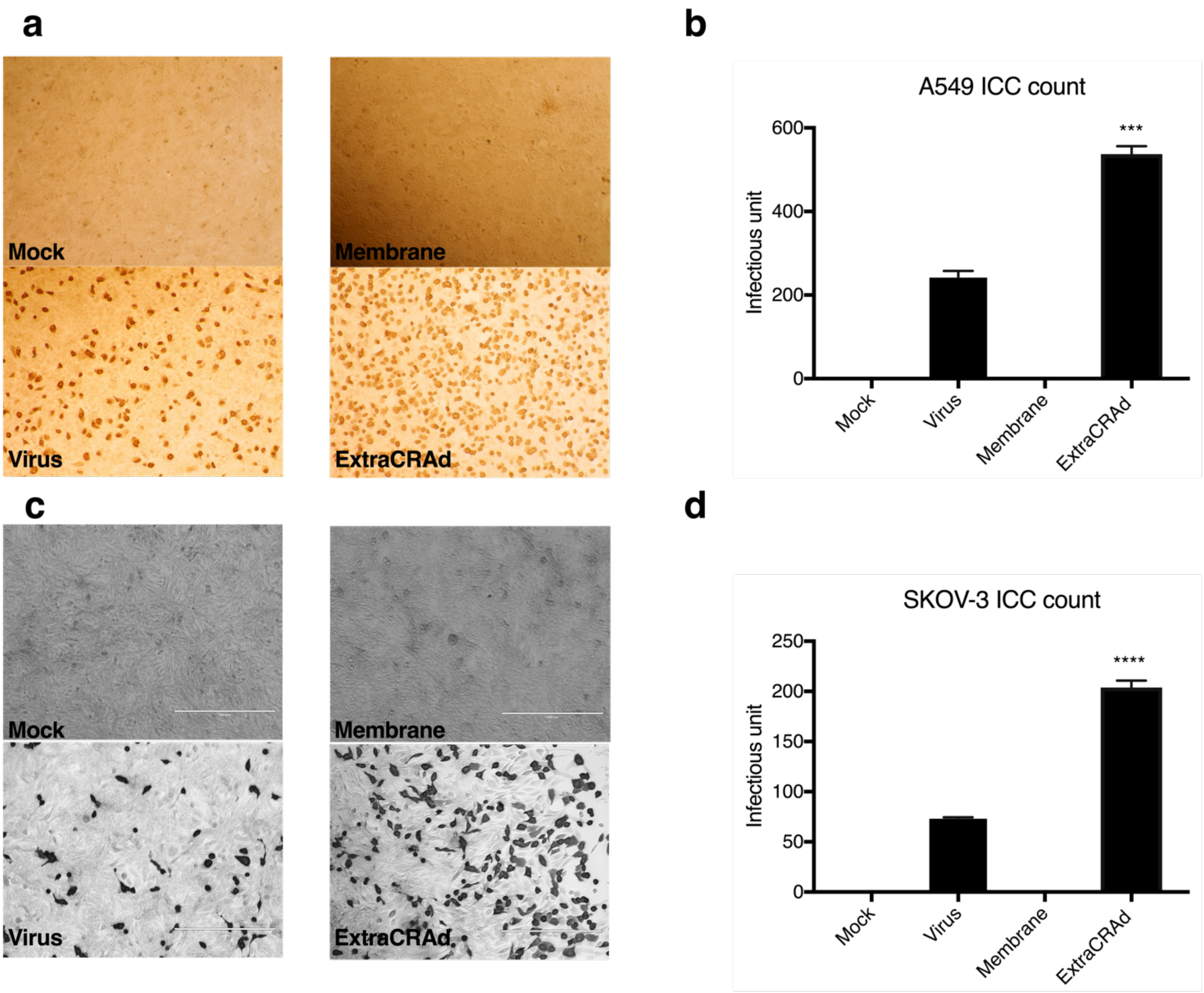


**Supplementary Figure 5. Increased infectivity by ExtraCRAd technology**. Immuno-cyto-chemistry for monoclonal antibody against Hexon protein (black spots). (**a**)Intracellular staining for high CAR human lung cancer cell line A549. (**b**) Infectious unit defined by spots detected through immuno-cyto-chemistry. (**c**) Intracellular staining of adenoviral Hexon protein for human ovarian cancer cell line SKOV-3 bearing low CAR receptor. (**d**) Infectious unit quantification (spots counted).

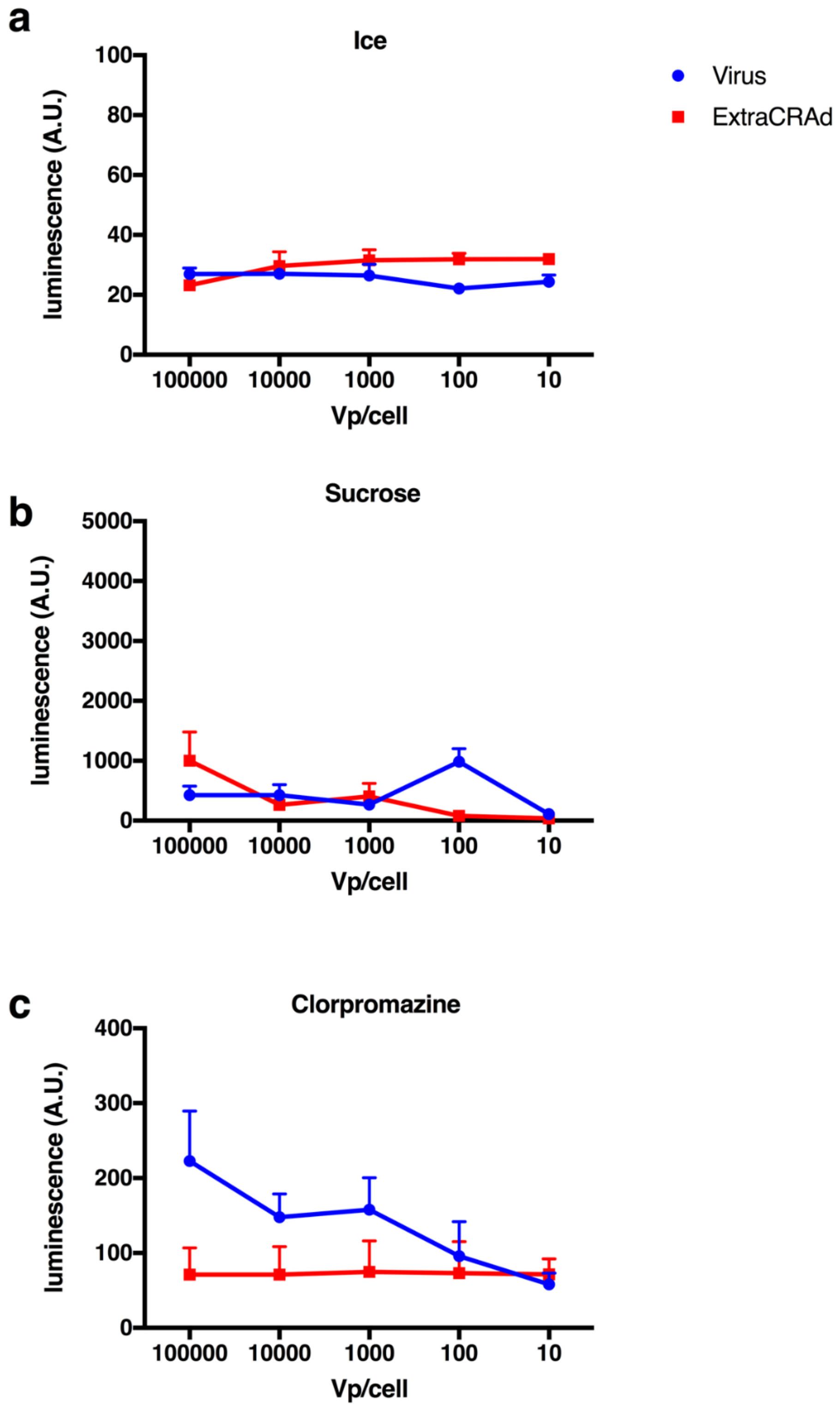


**Supplementary Figure 6.** Investigation of the uptake mechanism: (**a**) evaluation of the contribution from active and passive uptake on ice, and investigation of the endocytosis pathway by pre-incubation with (**b**) sucrose and (**c**) chlorpromazine. The cells were infected with ExtraCRAd or the naked virus

for 2 h, at different ratios of viral particles (vp) per cell, followed by 24 h of incubation, before being lysed. The data are presented as mean ± s.d. ($n \geq 3$). The data were analysed by two-way ANOVA, followed by Sidak post-test.

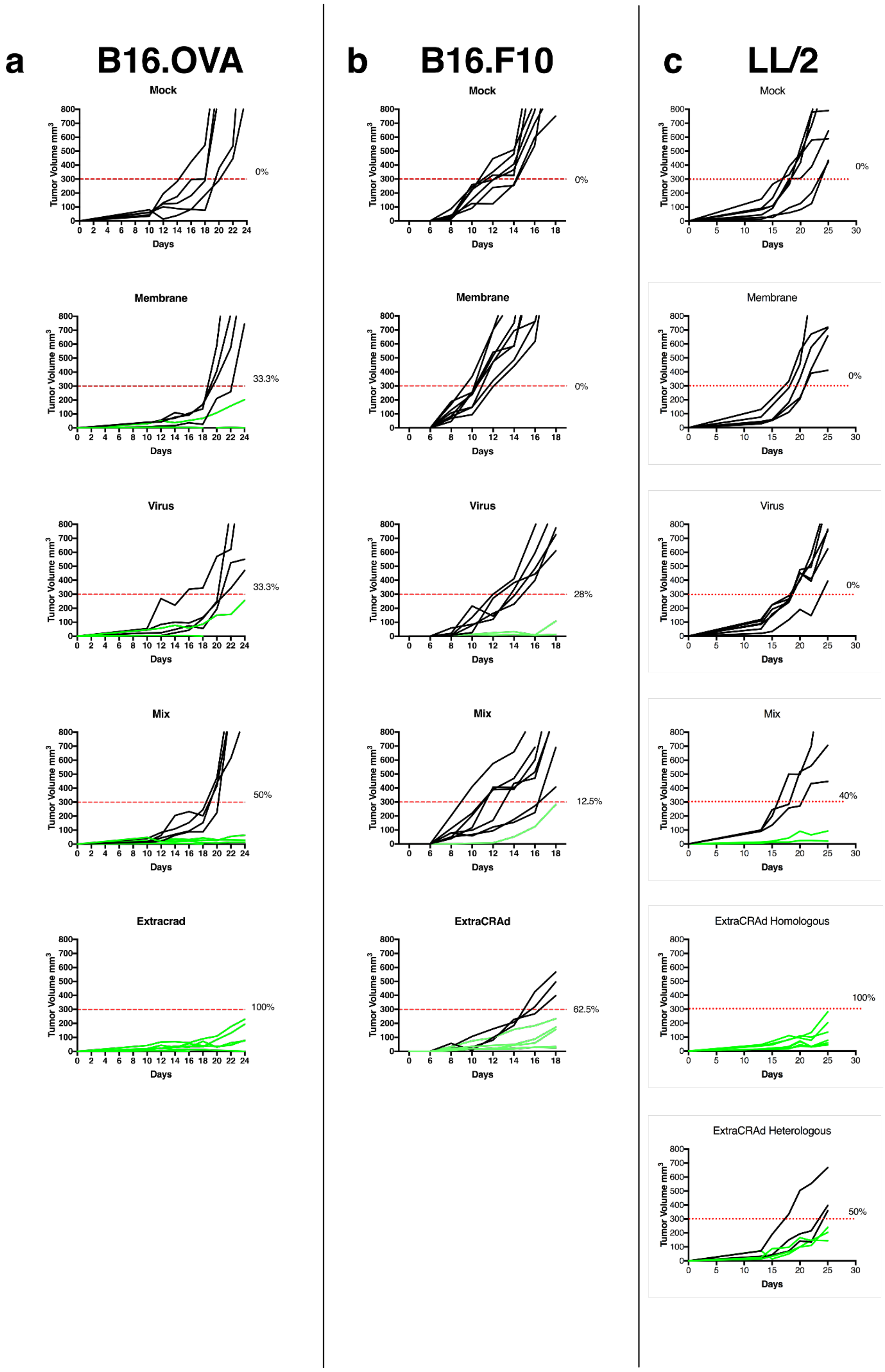


**Supplementary Figure 7.** Single tumor growth curves for (**a**) B16.OVA, (**b**) B16.F10, and (**c**) LL/2 tumor models. In the single mouse curves, the responders to the therapy are highlighted in green. The

threshold for responders was set at 300 $mm^3$ (red scattered line). The percentage of responders was calculated dividing the number of responders for the total number of animals in the group.

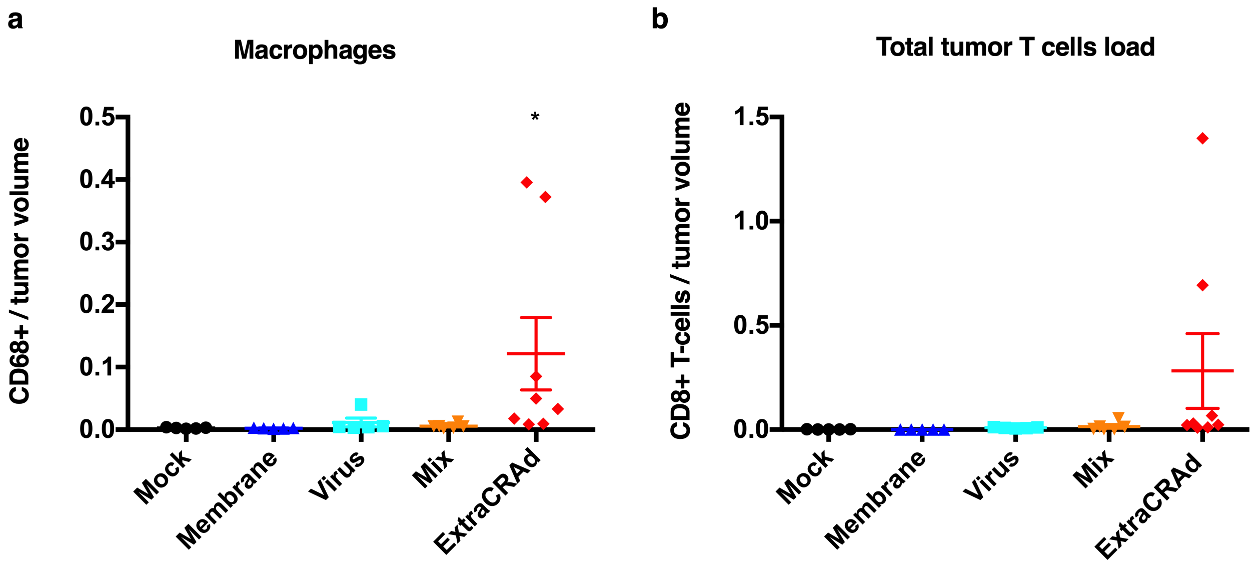


**Supplementary Figure 8. Immunological analyses of tumor microenvironment in B16F10 melanoma mice model.** (**a**) $CD68^+$ macrophages present within the tumor; (**b**) $CD8^+$ T-cells within the tumor. Mix identifies a treatment composed of cell membrane vesicles just mixed with adenovirus. The data are presented as mean ± s.d ($n \geq 5$), together with the dot plot of the single value. The data were analyzed with two-way ANOVA, followed by Fisher LSD post-test. The level of significance was set at the probability of $*p<0.05$.

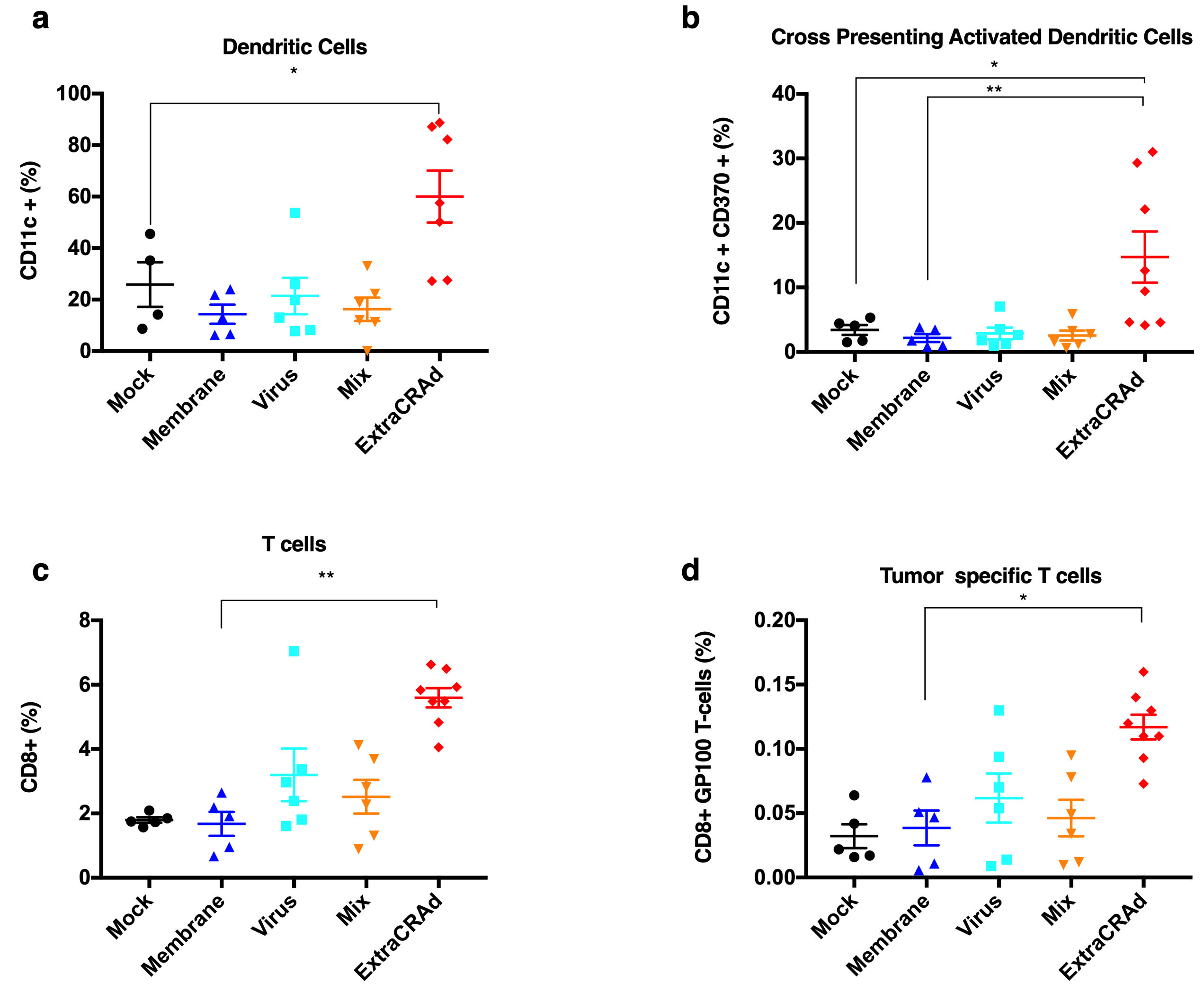


**Supplementary Figure 9. Immunological analysis of spleen in B16F10 tumor model**. (**a**) Percentage of CD11c$^{+}$ dendritic cells; (**b**) percentage of CD370$^{+}$ cross-presenting dendritic cells in the spleen of the animals. (**c**) Percentage of CD8+ T-cells in the spleen of the animals and (**d**) gp100 pentamer-specific T-cells, identifying the tumor-specific T-cells in the spleen of the animals. Mix identifies a treatment composed of cell membrane vesicles just mixed with adenovirus. The data are presented as mean ± s.d ($n \geq 5$), together with the dot plot of the single value. The data were analyzed with two-way ANOVA, followed by Dunnet's post-test. The levels of significance were set at the probabilities of *$p<0.05$ and **$p<0.01$.

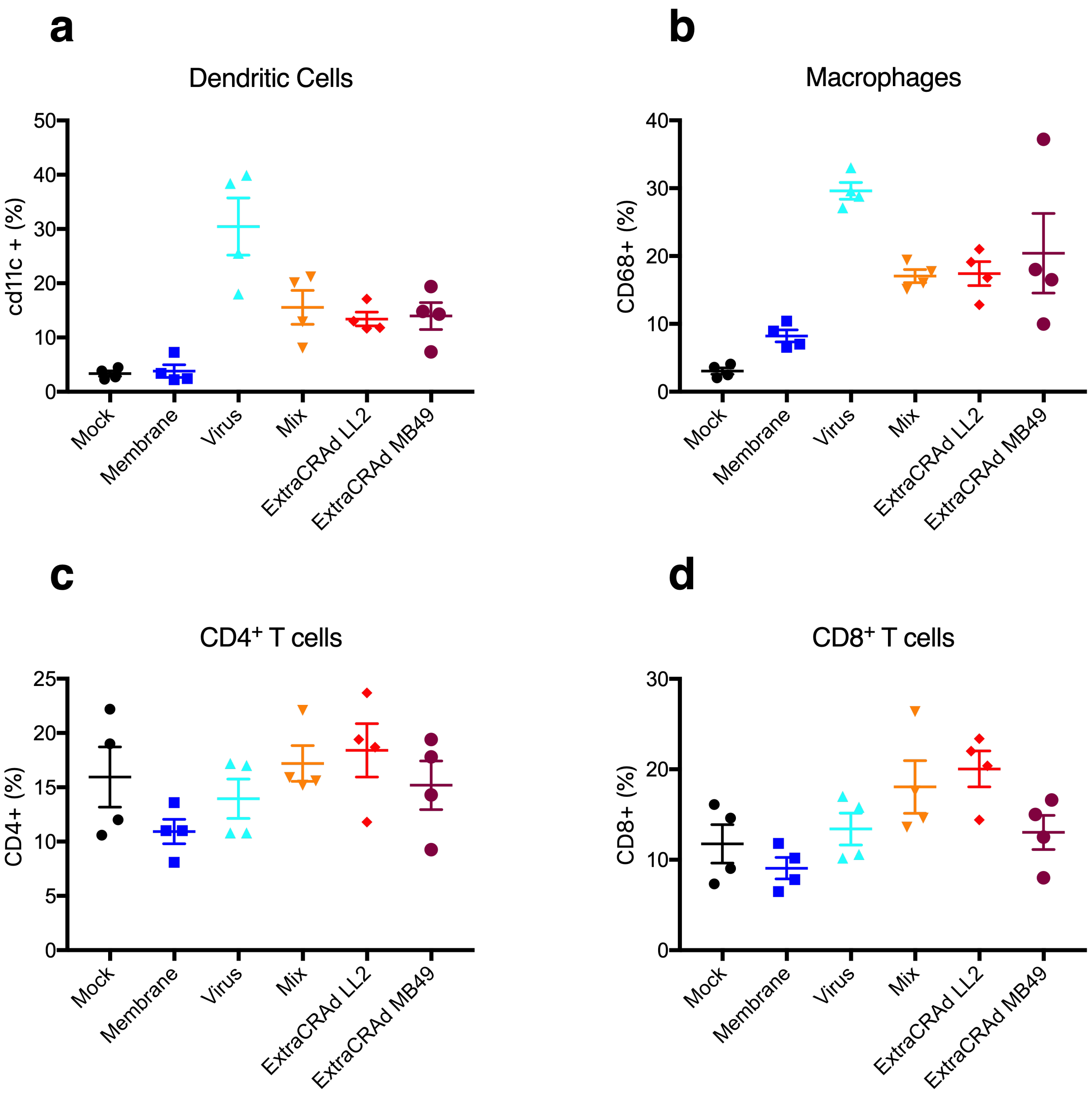


**Supplementary Figure 10. Immunological analysis of spleen in LL/2 tumor model.** (**a**) Percentage of CD11c$^+$ dendritic cells; (**b**) percentage of CD68$^+$ macrophages in the spleen. (**c**) Percentage of CD4$^+$ T-cells in the spleen and (**d**) percentage of CD8$^+$ T-cells. Mix identifies a treatment composed of cell membrane vesicles just mixed with adenovirus. The data are presented as mean ± s.d (*n=4*), together with the dot plot of the single value. The data were analyzed with two-way ANOVA, followed by Dunnet's post-test.

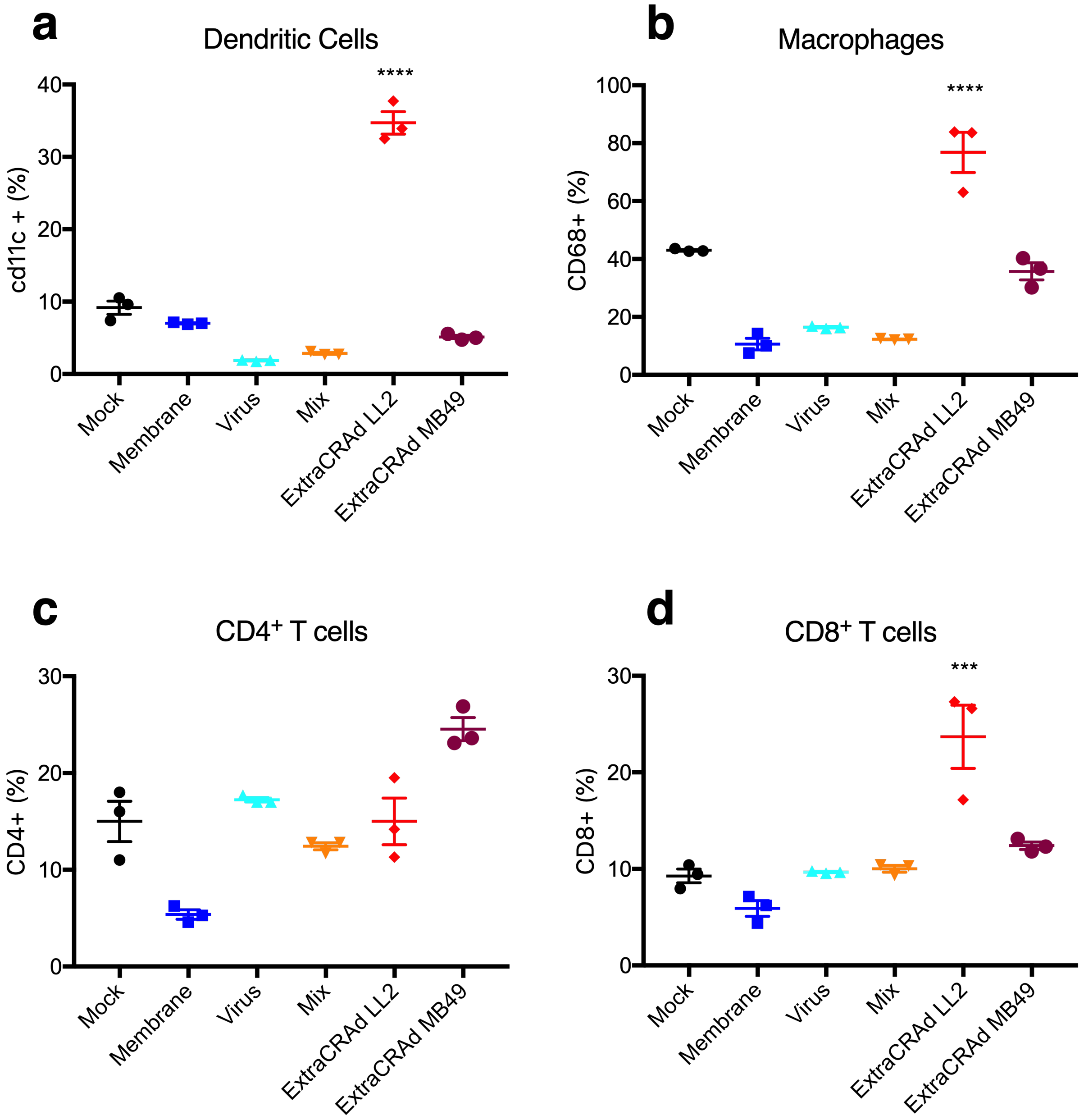


**Supplementary Figure 11. Immunological analysis of tumor draining lymph nodes in LL/2 tumor models.** (**a**) total dendritic cells in the lymph nodes ($CD11c^+$ cells); (**b**) total macrophage population in the lymph nodes ($CD68^+$ cells); (**c**) T-helper T cells ($CD4^+$ cells); (**d**) cytotoxic T cells ($CD8^+$ cells). The results are presented as mean±s.d. (*n=3*). The data were analyzed with one-way ANOVA followed by Dunnet's post-test comparison. The levels of statistical significance were set at probabilities ***$p<0.001$ and ****$p<0.0001$.

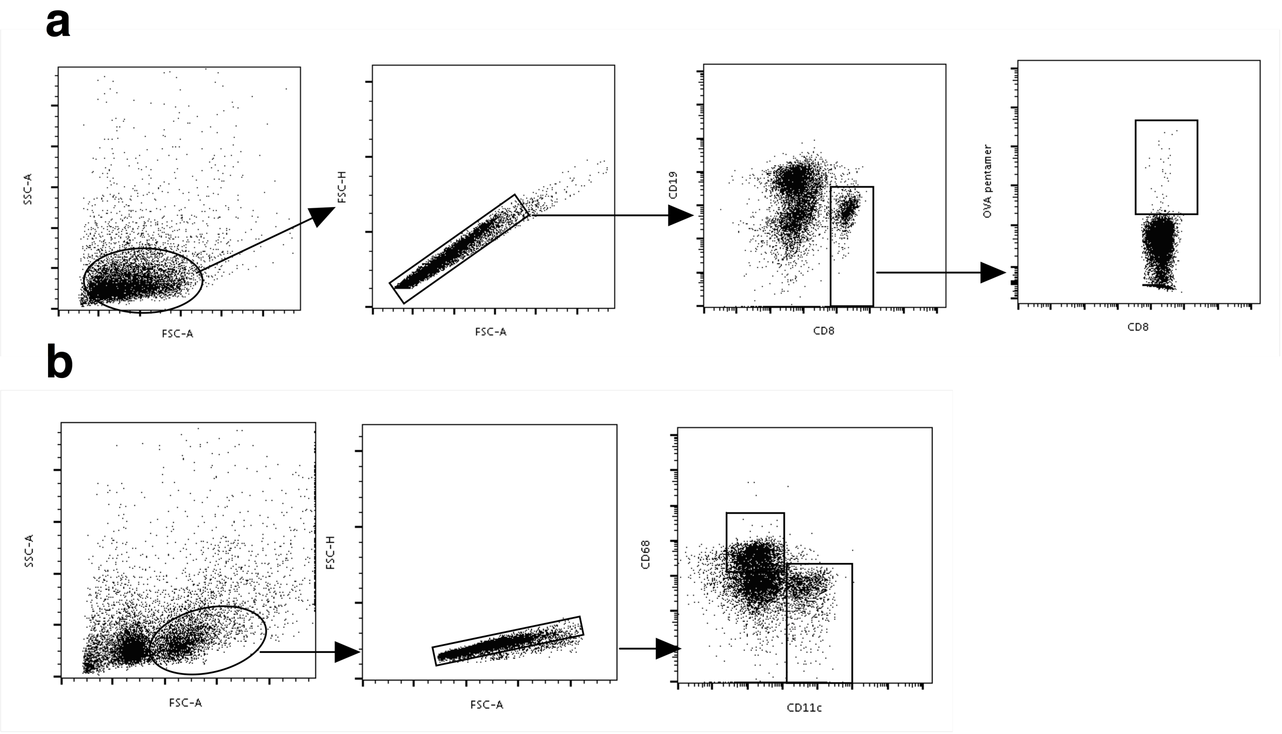


**Supplementary Figure 12. Gating strategy used for cell sorting.** (**a**) Gating strategy to sort OVA specific T lymphocytes (CD19-CD8+SIINFEKL+) from ex vivo tissues. (**b**) Gating strategy to sort antigen presenting cells, CD68 for macrophages and CD11c for dendritic cells of ex vivo tissues.
