# Supplementary material for: Artificially cloaked viral nanovaccine for cancer immunotherapy
Source: Nat Commun. 2019 Dec 17;10:5747. doi: 10.1038/s41467-019-13744-8 (PMC6917704; doi:10.1038/s41467-019-13744-8)
Supplement: Supplementary file 3 — Reporting Summary [file 41467_2019_13744_MOESM3_ESM.pdf]

nature research

Corresponding author(s): Vincenzo Cerullo

Last updated by author(s): Nov 14, 2019

Reporting Summary

Nature Research wishes to improve the reproducibility of the work that we publish. This form provides structure for consistency and transparency in reporting. For further information on Nature Research policies, see Authors & Referees and the Editorial Policy Checklist .

Statistics

For all statistical analyses, confirm that the following items are present in the figure legend, table legend, main text, or Methods section.

n/a	Confirmed	
☐	☒	The exact sample size (*n*) for each experimental group/condition, given as a discrete number and unit of measurement
☐	☒	A statement on whether measurements were taken from distinct samples or whether the same sample was measured repeatedly
☐	☒	The statistical test(s) used AND whether they are one- or two-sided<br>*Only common tests should be described solely by name; describe more complex techniques in the Methods section.*
☒	☐	A description of all covariates tested
☒	☐	A description of any assumptions or corrections, such as tests of normality and adjustment for multiple comparisons
☐	☒	A full description of the statistical parameters including central tendency (e.g. means) or other basic estimates (e.g. regression coefficient) AND variation (e.g. standard deviation) or associated estimates of uncertainty (e.g. confidence intervals)
☐	☒	For null hypothesis testing, the test statistic (e.g. *F*, *t*, *r*) with confidence intervals, effect sizes, degrees of freedom and *P* value noted<br>*Give P values as exact values whenever suitable.*
☒	☐	For Bayesian analysis, information on the choice of priors and Markov chain Monte Carlo settings
☒	☐	For hierarchical and complex designs, identification of the appropriate level for tests and full reporting of outcomes
☒	☐	Estimates of effect sizes (e.g. Cohen's *d*, Pearson's *r*), indicating how they were calculated

Our web collection on statistics for biologists contains articles on many of the points above.

Software and code

Policy information about availability of computer code

Data collection	None
Data analysis	For data analysis and figure generation Graphpad Prism was employed

For manuscripts utilizing custom algorithms or software that are central to the research but not yet described in published literature, software must be made available to editors/reviewers. We strongly encourage code deposition in a community repository (e.g. GitHub). See the Nature Research guidelines for submitting code & software for further information.

Data

Policy information about availability of data

All manuscripts must include a data availability statement. This statement should provide the following information, where applicable:

- Accession codes, unique identifiers, or web links for publicly available datasets
- A list of figures that have associated raw data
- A description of any restrictions on data availability

Data available on request from the authors

Field-specific reporting

Please select the one below that is the best fit for your research. If you are not sure, read the appropriate sections before making your selection.

☒ Life sciences ☐ Behavioural & social sciences ☐ Ecological, evolutionary & environmental sciences

For a reference copy of the document with all sections, see nature.com/documents/nr-reporting-summary-flat.pdf

Life sciences study design

All studies must disclose on these points even when the disclosure is negative.

Sample size	All in vitro data were done at least in triplicate and SE or SD were reported as stated in the figure and figure legends. The animal studies were always conducted with a sample size of at least 8 mice per group 1 tumor per mouse as indicated in our material and methods.
Data exclusions	DATA were not excluded. Some mice have been excluded before the randomization if tumors were not of the right injectable size.
Replication	For ethical reason we did not repeat the same exact experiment, we have however validated our DATA by repeating the experiment with different tumor model and by increasing the size of the cohorts.
Randomization	Mice were blindly randomized by Lab technicians or the students carrying out the experiments.
Blinding	As general practice in our Lab the student that is measuring the tumor does not know what cage (treatment) is measuring. In this way all the measurement are always done blindly.

Reporting for specific materials, systems and methods

We require information from authors about some types of materials, experimental systems and methods used in many studies. Here, indicate whether each material, system or method listed is relevant to your study. If you are not sure if a list item applies to your research, read the appropriate section before selecting a response.

Materials & experimental systems

n/a	Involved in the study	
☐	☒	Antibodies
☐	☒	Eukaryotic cell lines
☒	☐	Palaeontology
☐	☒	Animals and other organisms
☒	☐	Human research participants
☒	☐	Clinical data

Methods

n/a	Involved in the study	
☒	☐	ChIP-seq
☐	☒	Flow cytometry
☒	☐	MRI-based neuroimaging

Antibodies

Antibodies used

Anti Adenovirus type 5 antibody (rabbit polyclonal), Abcam, catalog n.ab6982, batch .GR127734-4
FC-block CD16/2 antibody, True stain fcX clone 93, Biolegend, catalog n.101320, batch n.B254979
FITC-antiCD8, clone KT15, rat antimouse IgG2a, Proimmune, catalog n.A5402-3bE, batch n.1603F/29629
PE-antiPD1 (CD279), clone 29F.1A12, BIolegend, catalog n. 135206, batch n.B210494
PE-Cy7-anti CD19, clone 6D5, Biolegend, catalog n. 115520, batch n. B201235
APC-MHC Class I Pentamer (H-2Kb), clone SF1.1.1, Biolegend, catalog n. 116619, batch n. B216126
FITC-antiCD11c, clone N418, Biolegend, catalog n. 117306, batch n. B202912
PE-anti370 (CLEC9A-DNGR1), clone 7H11, Biolegend, catalog n. 143504, batch n. B178553
PEr-CP-anti CD86, clone GL-1, Biolegend, catalog n. 105028, batch n. B251107
APC-anti H-2Kb bound to SIINFEKL, clone 25-D1.16, Biolegend, catalog n, 141606, batch n. B200632
PE-Cy7-anti CD 19, clone 6D5, BIolegend, catalog n. 115520
APC-anti CD8 SIINFEKL, Proimmune, catalog n. F093-84C-E, batch n. OP/5821-03
PE-anti gp100 Pentamer, Proimmune, cotalog n.F2296-2B, Batch n. PP/6269-16
APC-anti TRP2 Pentamer, Proimmune, catolg n. F185-4B-185, batch n. OP/5821-03
APC-anti PD1, clone 29F.1A12, Biolegend, catalog n. 135209, batch n.B239832
CD68, clone FA-11, Biolegend, catalog n. 137013, batch n. B192561
APC-anti CD4, clone GK1.5, Biolegend, catalog n.100412, batch n.

Validation

Abcam ab6982 is a polyclonal antibody against Purified Adenovirus type 5. This antibody has been validated by the producer for the application (ELISA) used in this study. Please find data sheet from the producer here: https://www.abcam.com/adenovirus-type-5-antibody-ab6982-references-html.
Fc block CD16/2 antibody, True stain fcX clone 93, Biolegend is a rat IgG2a lambda antimouse antibody. The validation has been performed by the manufacturer: https://www.biolegend.com/en-us/global-elements/pdf-popup/trustain-fcx-anti-mouse-cd16-32-antibody-5683?filename=TruStain%FcX%20anti-mouse%20CD1632%20Antibody-pdf&pdfgen=true.
PE-antiPD1 (CD279), clone 29F.1A12, Biolegend, catalog n. 135206 is a monoclonal rat IgG2a k antimouse antibody. The product was validated by the manufacturer: https://www.biolegend.com/en-us/global-elements/pdf-popup/pe-anti-mouse-cd279-pd-1-antibody-6179?filename=PE%20CD279%20PD-1%20Antibody.pdf&pdfgen=true.
Proimmune F2296-2B, F185-4B-185, and F093-84C-E are pentamers against specific peptides developed by Proimmune. The antibodies have been validated for the application (flow cytometer) by the manufacturer. Please find the validation from the

producer here: https://www.proimmune.com/eccomerce/pdf_files/PS_F-APC_V1.1%20%28Pro5%C2%AE%20MHC%20Class%20I%20Pentamer%20%28APC%20Labeled%29%29.pdf.
PE-Cy7 CD19 from Biolegend is a monoclonal antibody rat IgG2a, k antimouse. The antibody has been validated by the manufacturer. Please find the validation here: https://www.biolegend.com/en-us/global-elements/pdf-popup/pe-cy7-anti-mouse-cd19-antibody-1907?filename=PECy7%20anitmouse%20CD19%20Antibody.pdf&pdfgen=true.
APC-MHC Class I Pentamer (H-2kb), clone SF1.1.1 Biolegend, catalog n.116619 is a mouse (SJL) IgG2a, K antibody. The product was validated by the manufacturer: https://www.biolegend.com/en-us/global-elements/pdf-popup/apc-anti-mouse-h-2kd-antibody-6845?filename=APC%20anti-mouse%20H-2KSUPdSUP%20Antibody.pdf%pdfgem=true.
FITC-antiCD11c, clone N418, Biolegend, catalog n. 117306, is an Armenian Hamster IgG anti mouse antibody. The product was validated by the manufacturer: https://www.biolegend.com/en-us/global-elements/pdf-popup/fitc-anti-mouse-cd11c-antibody.1815?filename=FITC%20anti-mouse%20CD11c%20Antibody.pdf&pdfgen=true.
PE-anti Cd370 (CLEC9A-DNGR1), clone 7H11, Biolegend, catalog n. 143504, is a rat IgG1, k anti mouse antibody. The product was validated by the manufacturer: https://www.biolegend.com/en-us/global-elements/pdf-popup/pe-anti-mouse-cd370-clec9a-dngr1-antibody-7689?filename=PE%20anti-mouse%20CD370%20CLEC9A%20DNGR1%20Antibody.pdf&pdfgen=true.
Per-CP- anti CD86, clone GL-1, BIolegend, catalog n. 105028, is a rat IgG2a, k antimouse monoclonal antibody. The product was validated by the manufacturer: https://www.biolegend.com/en-us/global-elements/pdf-popup/percpcyanine55-anti-mouse-cd86-antibody-4276?filename=PerCPCyanine55%20anti.mouse%20CD86%20Antibody.pdf&pdfgen=true.
APC-anti H-2kb bound to SIINFEKL, clone 25-D1.16, Biolegend, catalog n. 141606 is a monoclonal mouse IgG1. The product was validated by the maufacturer: https://www.biolegend.com/en-us/global-elements/pdf-popup/apc-anti.mouse-h-2kb-bound-to-siinfekl-antibody-7882?filename=APC%20anti-mouse%20H-2Ksupbsup%20bound%20to%20SIINFEKL%20Antibody.pdf&pdfgen=true.
APC-anti PD1, clone 29F.1A12, Biolegend, catalog n. 135209, is a rat IgG2a, k monoclonal antimouse antibody. The product was validated by the manufacturer: https://www.biolegend.com/en-us/global-elements/pdf-popup/apc-anti-mouse-cd279-pd-1antibody-6497?filename=APC%20CD279%20PD-1%20Antibody.pdf&pdfgen=true.
APC-antimouse CD4, Biolegend, catalog n.100516, is a Rat IgG2a, K antimouse antibody. The validation was performed by the provider:https://www.biolegend.com/en-us/global-elements/pdf-popup/apc-anti-mouse-cd4-antibody-245?filename=APC%20anti-mouse%20CD4%20Antibody.pdf&pdfgen=true

Eukaryotic cell lines

Policy information about cell lines

Cell line source(s)	A549 cells, LL-2 cells, and B16.F10 cells were purchased from the American Type Culture Collection (ATCC; Manassas, VA, USA). The mouse melanoma cell line B16.OVA expressing chicken OVA was supplied by Prof. Richard Vile (Mayo Clinic, Rochester, MN, USA) and the human ovarian cancer cell line SKOV-3 LUC expressing luciferase was provided by Dr. Negrin (Stanford Medical School, Stanford, CA, USA). CMT64.OVA were a kind gift from Dr. Florian Kuhnel (Hannover Medical School, Hannover, Germany)
Authentication	None of the cell lines were authenticated in house after 2015.
Mycoplasma contamination	All the cell lines have been tested for mycoplasma with MycoAlertTM mycoplasma detection kit by Lonza (LT07-318) and the results of the tests were negative.
Commonly misidentified lines (See ICLAC register)	*Name any commonly misidentified cell lines used in the study and provide a rationale for their use.*

Animals and other organisms

Policy information about studies involving animals; ARRIVE guidelines recommended for reporting animal research

Laboratory animals	Immunocompetent female 4-6 weeks old, C57BL/6JOlaHsd mice obtained by ENVIGO C57BL/6.<br>Immunodeficient female 4-6 week old HsdCpb:NMRI-Foxn1nu nude mice obtained by ENVIGO.
Wild animals	The study did not involve wild animals.
Field-collected samples	The study did not involve collecting samples in the field.
Ethics oversight	The animal experiments were approved by Animal Experimental Board (ELLA board) of Southern Finland. Moreover, all the animal experiments were approved by the laboratory animal center veterinarian.

Note that full information on the approval of the study protocol must also be provided in the manuscript.

Flow Cytometry

Plots

Confirm that:

- [x] The axis labels state the marker and fluorochrome used (e.g. CD4-FITC).
- [x] The axis scales are clearly visible. Include numbers along axes only for bottom left plot of group (a 'group' is an analysis of identical markers).
- [x] All plots are contour plots with outliers or pseudocolor plots.
- [x] A numerical value for number of cells or percentage (with statistics) is provided.

Methodology

Sample preparation	The cells analyzed by FACS were derived from the in vivo experiments. In detail, at the end of each animal experiment, lymphoid organs as tumor, spleens, and lymph nodes per each mouse were collected into 50 mL falcon tubes filled with 10%of FBS RPMI medium. The tubes were kept on ice until the organs were smashed. All the organs were smashed using a cell strainer (70 and 40 um mesh, BD Biosciences, USA). The cell suspension was supplemented with 10% DMSO, aliquoted in cryovials and stored at -80° C. After thawing splenocytes and lymphocytes for each sample, 200 ul of cell suspension were transferred from the cryovial into 96-well V-bottom plates. In the case of tumor samples, only 100 ul were used instead of 200. The plate was centrifuged for 5 min at 2000 rpm, at 4°C, the supernatant was discarded and the cell pellet was washed twice with PBS. The cell pellet was then resuspended in 40 ul of TruStainFcXtm (antimouse Cd16/32, product number 101320, Biolegend) Fc bloc antibody diluited 1:20 with PBS per well. After 15 minutes of incubation at 4°C, pentamer antibody by Proimmune was added (20 uL) to each well while PBS 1X was added to the unstained ones. The samples were incubated for 20 min in the dark at room temperature. The samples were then washed twice with 100 ul of PBS and centrifuged for 5 min at 2000 rpm at 4°C. The supernatant was discared and the pellet was resuspended in 50 ul of antibodies cocktail (for the other stainings). Controls and unstained samples were resuspended in 50 ul of PBS and then incubated for 30 min at +4°C in the dark. After incubations, the cells were washed twice with 100 ul of PBS and centrifuged for 5 min at 2000 rpm. Finally, the samples were resuspended in 180 ul of PBS and directly read with the flow cytometer BD Accuri tm C6Plus.
Instrument	All the samples were analyzed with a Bd Accuri flow cytometer, equipped with a C6 automatic samples. The manufacturer is BD Biosciences (USA).
Software	The data were collected with BD Accuri flow cyotometer C6 software (BD Biosciences, USA) and the FCS files were then analyzed with FlowJo v.10 (Tree Star, Ashland, OR, USA).
Cell population abundance	5x 10^5 raw events were acquired for each sample. Cell debris and dead cells were excluded. Within the popolation of interest, 2 x 10^5 events were acquired.
Gating strategy	Cell population was first cleaned by eliminating debris and dead cells. Live cells were gated and doublets excluded. Within that live population, the lymphocyte population was identified and gated. Then CD19 positive cells were excluded. The CD8 population was investigated with the markers of interest. As for the APC, the CD11c positive cells were gated and further analyzed with the markers of interest.

- [x] Tick this box to confirm that a figure exemplifying the gating strategy is provided in the Supplementary Information.
